# Impact of face masks on empathy and communication in head and neck cancer patients: a case-control study

**DOI:** 10.3389/fonc.2025.1539070

**Published:** 2025-03-07

**Authors:** Georg Hoene, Nikolaus von Hahn, Tim Mathea, Boris Schminke, Kathi Goldstein, Martin Leu, Henning Schliephake, Susanne Wolfer

**Affiliations:** ^1^ Clinic for Oral and Maxillofacial Surgery, University Medical Center Goettingen, Goettingen, Germany; ^2^ Department of Radiotherapy and Radiation Oncology, University Medical Center Goettingen, Goettingen, Germany

**Keywords:** oral squamous cell carcinoma, COVID 19, face masks, CARE-Questionnaire, empathy, physician-patient relations, communication barriers, oral and maxillofacial surgery

## Abstract

**Background:**

The COVID-19 pandemic necessitated the widespread use of face masks in medical settings. In the treatment of patients with head and neck tumors or other malignancies, where non-verbal communication and emotional expression are critical, face masks may potentially impair the physician-patient relationship. This study aimed to assess the impact of face masks on perceived empathy and the quality of physician-patient communication in this patient cohort.

**Methods:**

A prospective case-control study as part of the postoperative follow-up consultations was conducted at the Department of Oral and Maxillofacial Surgery, University Medical Center Goettingen, utilizing the Consultation and Relational Empathy (CARE) measure. Patients were divided into two groups: in the case group, clinicians wore face masks throughout the entire consultation, while in the control group, masks were worn only during the clinical examination. The primary outcome was the difference in CARE scores, reflecting the perceived empathy of the clinician.

**Results:**

No significant difference in mean CARE scores was observed between the two groups (p=0.454), indicating that wearing a face mask did not significantly affect patients’ perception of clinician empathy. However, a significant positive correlation was found between patients’ familiarity with the clinician and higher CARE scores (p=0.003). Other variables, such as patient health status and consultation duration, did not significantly influence CARE scores.

**Conclusion:**

Wearing face masks does not significantly impair perceived empathy in the context of physician-patient communication. Familiarity with the clinician emerged as a key factor in enhancing the quality of the interaction. These findings underscore the importance of fostering long-term, trust-based physician-patient relationships, particularly during periods of pandemic-related protective measures such as mask mandates.

## 1 Introduction

Effective communication between physician and their patients is a fundamental aspect of clinical practice (1). Particularly the lower part of the face is of importance in terms of its role in the expression of emotions and moods (2, 3). The use of a face mask covering the entire lower face of the clinician has been shown to impair and distort the perception of certain emotions (4, 5). Additionally, wearing a face mask has been demonstrated to negatively impact communication, further complicating the physician-patient interaction (6, 7).

In response to the alarming rise in SARS-CoV-2 infection rates, the federal government enacted a nationwide mask mandate at the end of April 2020 in Germany (8). The wearing of a face mask constituted an essential component of the protection measures implemented in response to the Coronavirus, both in public spaces and in medical facilities. While medical face masks (such as surgical masks) serve primarily to protect others from the droplets of exhaled air produced by the wearer (external protection), particle-filtering half masks (such as FFP2 and FFP3 masks) are also capable of protecting the wearer from infection (self-protection) (9). Masks were a mandatory requirement at the Goettingen University Medical Center from the inception of the measures in April 2020 until April 2023. This was implemented with due diligence by both medical staff and patients (10).

The efficacy of face masks in preventing the transmission of SARS-CoV-2 has been substantiated by several studies (11–13) They demonstrated that the use of face masks can reduce the risk of infection with SARS-CoV-2 in medical staff by up to 70%. In contrast with the evidence supporting the efficacy of face masks, the requirement to wear them has been a topic of ongoing controversy and political debate (14). A number of factors have been identified as influencing the acceptance of mandatory face masks, including socioeconomic and psychological characteristics, risk perception of the pandemic, empathy, trust in healthcare professionals and political attitudes (15, 16).

The existing literature also identifies several adverse effects associated with mask wearing. These include voice fatigue (17), eye dryness (18), dermatological diseases (19), discomfort (20) and obstruction of oxygen exchange (21).

Furthermore, the use of face masks has been found to have a detrimental impact on interpersonal communication, which is a crucial aspect of the physician-patient relationship. Studies have indicated that wearing a face mask can impede understanding speech (22), diminish the quality of medical consultations (23) and impair emotion recognition accuracy as well as perceived closeness (6). The reduction in speech volume, the inability to lip-read, and the dampening of certain sound frequencies result in significant communication difficulties, particularly for patients with hearing loss. Additionally, the absence of visual cues further exacerbates these challenges, especially in noisy environments (24–26).

The facial region plays a pivotal role in the expression of emotions and moods. In terms of the recognition of different emotions, the existing literature indicates that the mouth region is of particular importance for the recognition of happiness, neutral expressions and anger (2, 3, 27). In their study, Tsantani et al. (2022) demonstrated that wearing a face mask results in a reduction in the perceived intensity of intended emotions and an increase in the perceived intensity of unintended emotions (5). In general, individuals tend to have greater difficulty correctly categorizing the emotions of mask wearers (28). The perception of the emotion anger is not influenced by the act of wearing a face mask (5, 29).

A distinctive instrument utilized in the assessment of patient-centered care is the Consultation and Relational Empathy (CARE) Measure. The instrument was developed in 2004 by Mercer and coworkers at the Departments of General Practice at the Universities of Glasgow and Edinburgh. It is a measuring instrument that records the clinicians clinical empathy from the patient’s perspective. The CARE questionnaire allows patients to assess the ‘human’ aspects of their consultations, offering direct feedback to clinicians on the strengths and weaknesses of their empathy. Its robust internal consistency and reliability have been validated across multiple languages and diverse clinical contexts. Built on a solid theoretical and empirical foundation, the questionnaire is applicable to a broad spectrum of diseases. As a result, our findings may be transferable to similar clinical settings, though additional validation is needed (30, 31). It has since been translated into numerous languages and utilized in diverse countries (32–38). This study employs the German version of the questionnaire, as presented by Neumann et al. (2008). Its robust internal consistency and reliability have been widely acknowledged and confirmed across different languages and clinical contexts.

In the studies conducted by Mercer and colleagues, the original version of the CARE questionnaire demonstrated high internal consistency, with Cronbach’s alpha coefficients ranging from 0.92 to 0.94 (30). Other authors have reported a higher internal consistency with a Cronbach’s alpha of 0.97 in their translated version (36, 38). Effective differentiation between the various clinicians is possible with only 15 to 20 patient ratings per clinician, provided that interrater reliability exceeds 0.8 (33).

In contrast to other measurement instruments, the CARE questionnaire is distinguished by its provision of additional explanations of specific clinician behaviors to respondents, utilizing synonymous and antonymous definitions (32).

Face masks are a critical component of infection control in oral and maxillofacial surgery, particularly during high-risk procedures, where minimizing transmission risks is paramount. Despite their clinical importance, limited research has examined their potential impact on physician-patient communication within this specialized setting. This study specifically aimed to assess whether wearing face masks influences the quality of communication and perceived empathy in patients with head and neck cancer. To the best of our knowledge, this represents the first investigation addressing these factors in the context of oral and maxillofacial surgery, thus filling a significant gap in the existing literature.

## 2 Materials and methods

This prospective, single-center case-control study was conducted at the Department of Oral and Maxillofacial Surgery, University Medical Center Goettingen, between November 14, 2023, and April 9, 2024, as part of postoperative follow-up consultations. The sample size calculation was based on key parameters from Mercer et al. (2005) and Mercer et al. (2011), which demonstrated the high internal consistency of the CARE questionnaire (Cronbach’s alpha 0.92–0.94) and its effectiveness in differentiating clinicians with 15–20 patient ratings per clinician (interrater reliability > 0.8) (31, 33). Using G*Power software (v. 3.1.9.6; University of Düsseldorf), the required minimum sample size was determined to be 122 patients (61 per group) based on a significance level of 0.05, a power of 0.95, and an effect size of 0.6.

### 2.1 Inclusion and exclusion criteria

Inclusion criteria comprised adult patients aged 18 years or older with the capacity to provide informed consent, undergoing postoperative follow-up consultations. Exclusion criteria included severe cognitive impairments, language barriers, or physical disabilities (e.g., vision or hearing loss) that could hinder study participation. Both groups included patients with tumors in the oral cavity, oropharynx, nasopharynx, hypopharynx, or facial region.

### 2.2 Group allocation

Patients were manually assigned to the case or control group in a structured manner using an alternating and consecutive method to ensure balanced distribution and minimize bias. In the case group, clinicians wore face masks throughout the entire consultation. In the control group, masks were used only during the direct clinical examination, adhering to standard practices for procedural sterility (hygiene), protection of vulnerable individuals from pathogen transmission (patient protection), and the broader reduction of infection spread within the clinical environment (infection control). This allocation approach ensured comparability between the groups while reflecting real-world clinical settings.

### 2.3 Clinical and pathological data

Clinical and pathological data were extracted from digital medical records to support exploratory analyses. Parameters included patient age, gender, tumor location, TNM stage, recurrence status, and surgical therapy. Additionally, clinician-related data, such as gender and mask usage, were documented.

### 2.4 Questionnaire and instrument

The study employed the German version of the Consultation and Relational Empathy (CARE) questionnaire, originally developed by Mercer et al. (2004) and translated by Neumann et al. (2008) (30, 32). The CARE questionnaire consists of ten items, rated on a 5-point Likert scale (strongly disagree to strongly agree), designed to measure the quality of consultations in terms of empathy, communication, and relational aspects. No modifications were made to the original instrument for this study.

### 2.5 Survey administration

The questionnaire was administered by a single interviewer to ensure consistency, and patient responses were anonymized to maintain confidentiality. After receiving an oral explanation of the study, patients signed an informed consent form. Responses were recorded either manually on paper and later digitized or directly via tablet or smartphone using a QR code. The questionnaire comprised three sections: (1) health status over the past two weeks, familiarity with the clinician, consultation duration, and face mask usage; (2) general attitudes and acceptance of face masks; and (3) feedback on consultations conducted with or without masks.

### 2.6 Ethical approval

The study was conducted in accordance with the tenets of the Declaration of Helsinki and reviewed and approved by a local ethics committee (vote number 33/8/23).

### 2.7 Statistical analysis

The statistical analysis was conducted using R (version 4.3.1; R Core Team, 2024). Since the Shapiro-Wilk test indicated that the data did not follow a normal distribution, the Mann-Whitney U test was used for group comparisons. Multiple linear regression analysis was performed to evaluate the relationships between independent variables (IVs) and the dependent variable (mean CARE scores), ensuring compliance with Gauss-Markov assumptions.

To enable comparability among IVs and assess their relative impact on the dependent variable, Z-standardization was applied. This transformation normalized the IVs to a mean of 0 and a standard deviation of 1, allowing for unit-independent comparison. All statistical tests were conducted at a significance level of α = 0.05.

**Table 1** summarizes the baseline characteristics of the patient cohort. The study included 93 men and 68 women, with a mean age of 69 years. Health status over the previous two weeks was rated as “acceptable” by 39% of patients, “satisfactory” by 37%, and “terrible” by only 2%.

**Table 1:** Patient Demographics and Clinical Characteristics (n = 166).

Category	Variable	n	%	Total
Sex (n = 166)	Male	93	56	166
	Female	68	44	
Age (n = 166)	Mean	69y	–	
	Standard Deviation	11.73y	–	
	Min-Max	34y–95y	–	
Health status (n = 161)	Terrible	3	2	161
	Bad	20	12	
	Acceptable	62	39	
	Satisfactory	59	37	
	Very good	17	11	
Type of tumor (n = 161)	Oral SCC	127	79	161
	Cutaneous SCC	12	7	
	Adenoid cystic carcinoma	5	3	
	Basal cell carcinoma	5	3	
	Salivary gland carcinoma	10	6	
	Others	2	1	
AJCC stage (n = 154)	Stage 0	7	5	154
	Stage I	62	40	
	Stage II	34	22	
	Stage III	23	15	
	Stage IVa	22	14	
	Stage IVb	5	3	
	Stage IVc	1	1	
	*Missing data (n = 2)*	–	–	
Tumor recurrence (n = 161)	Yes	20	12	161
	No	141	88	
Type of operation (n = 161)	Local flap	79	49	161
	Microvascular flap	75	46	
	Other	3	2	
	No surgery	4	3	

The majority of patients (79%) were diagnosed with oral squamous cell carcinoma (SCC), followed by cutaneous SCC (7%), salivary gland carcinoma (6%), adenoid cystic carcinoma (3%), and basal cell carcinoma (3%). Merkel cell carcinoma and lentigo malignant melanoma accounted for 1% of cases categorized as “other.” AJCC staging indicated that 66% of patients presented with early-stage tumors (Stages 0-II), while 34% were classified as advanced-stage (Stages III-IVC). AJCC staging was not applied to basal cell carcinoma cases. At the time of the study, 12% of the patient cohort had documented evidence of disease recurrence.

Most patients (97.5%) underwent primary surgical treatment, with only 2.5% receiving radiotherapy or chemotherapy as their primary modality. Among surgical cases, local defect closure was slightly more common than microvascular reconstruction. Microvascular techniques included the use of fibula, forearm flap, scapula, or anterolateral thigh (ALT) grafts, while local closure involved regional flaps such as submental island flaps, temporalis muscle flaps, nasolabial flaps, supraclavicular artery island flaps, or facial artery musculomuscular flaps. A small proportion of cases (2%) utilized full-thickness skin from the forearm, iliac crest, or omentum majus, classified as “other”.

## 3 Results

The specific characteristics relevant to the consultation are outlined in the subsequent sections. The patient cohort consisted of 161 individuals (93 male, 68 female) with a mean age of 69 years (SD = 11.73, range: 34–95). Most patients (79%) had oral squamous cell carcinoma, followed by cutaneous squamous cell carcinoma (7%), salivary gland carcinoma (6%), adenoid cystic carcinoma (3%), and basal cell carcinoma (3%). A small proportion of cases (2%) were categorized as “other,” including rare tumor types. Regarding tumor stage, 66% of patients presented with early-stage tumors (AJCC 0–II), whereas 34% were classified with advanced stages (III–IVc). Tumor recurrence was documented in 12% of the cohort.

Nine clinicians (six male, three female) participated, with face masks worn throughout consultations in 40% of cases (case group) and only during direct clinical examinations in 60% (control group).

**Figure 1:**
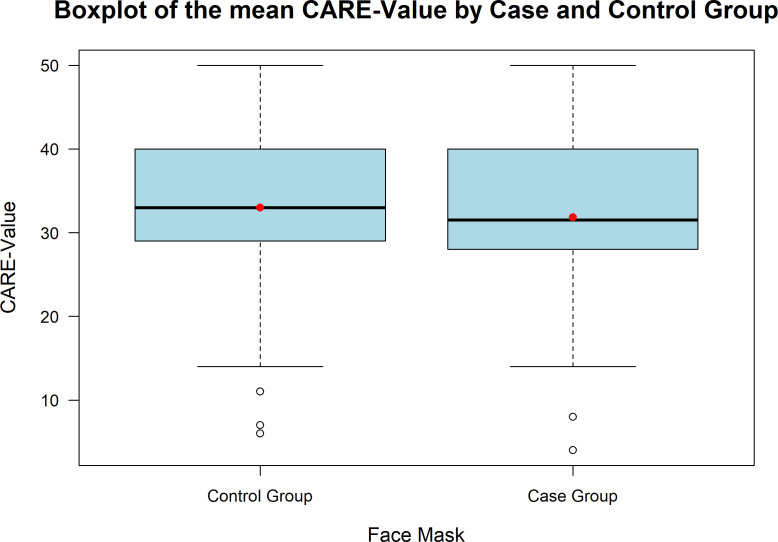
Boxplot of mean CARE scores for the case and control groups. Both groups show similar distributions, with a median CARE-Value around 35. The control group exhibits a slightly wider range and more outliers. The case group involved full face mask use during consultations, while the control group had partial usage.

The CARE score showed no significant differences between case and control groups (31.83 vs. 33.01; p = 0.454). The correlations are shown in **Figure 1**. Familiarity with the clinician varied among patients, with 38% indicating they knew the clinician “well,” 11% “very well,” 24% “not at all,” 5% “not well,” and 22% reporting a “neutral” level of familiarity. A significant positive correlation was observed between familiarity and higher CARE scores (p = 0.009). Neither tumor stage, recurrence status, nor consultation duration significantly influenced CARE values. Fifty percent of consultations lasted between 15 and 30 minutes, with max. 45 minutes. In the case group, a positive trend was observed, with CARE scores increasing as the consultation duration lengthened. However, this relationship was not evident in the control group. Overall, statistical analysis showed that consultation duration had no significant influence on CARE values (p = 0.32).

**Table 2** highlights the patients’ difficulties associated with the face mask, along with the reasons for these challenges. It is notable that only 5% of the patients reported feeling highly impaired by the face mask, while 76% of the patients indicated that they did not perceive any impairment. When queried about the underlying causes of this impairment, 21% of the patients indicated that they were unable to accurately identify the face and facial expressions of the clinician, while 73% of the patients did not perceive any of the provided response options to be an accurate representation of their experience. The variables presented in **Table 3** were answered by the entire patient population (n=161). The data indicates that only 11% of patients would prefer their clinician to wear a face mask for the entirety of the consultation. Most patients (48%) indicated that they would not prefer their clinician to wear a face mask for the entirety of the consultation. The question regarding the relevance of face masks in preventing the spread of respiratory infections in hospitals yielded less definitive responses. While 42% of the patients considered a face mask to be relevant, 32% were opposed to its use.

**Table 2:** Patient perception of impairment due to physicians wearing face masks (n = 66).

Variable	n (%)
Impairment by the face mask	
High	3 (4.55%)
Moderate	13 (19.70%)
Absent or None	50 (75.76%)
Total	66 (100%)
Reasons for the impairment caused by face masks	
Conversation is not properly understood	4 (6.06%)
Face and facial expressions are not recognized correctly	14 (21.21%)
Body language is not recognized correctly	0 (0%)
None of the answers match	48 (72.73%)
Total	66 (100%)

**Table 3:** Patients’ attitudes towards face masks (n = 161).

Variable	n (%)
Preference for a face mask	
Yes	17 (10.56%)
No	78 (48.45%)
No matter	66 (40.99%)
Total	161 (100%)
Relevance of a face mask to prevent respiratory infections	
Yes	68 (42.24%)
No	52 (32.30%)
No matter	41 (25.47%)
Total	161 (100%)

**Figure 2:**
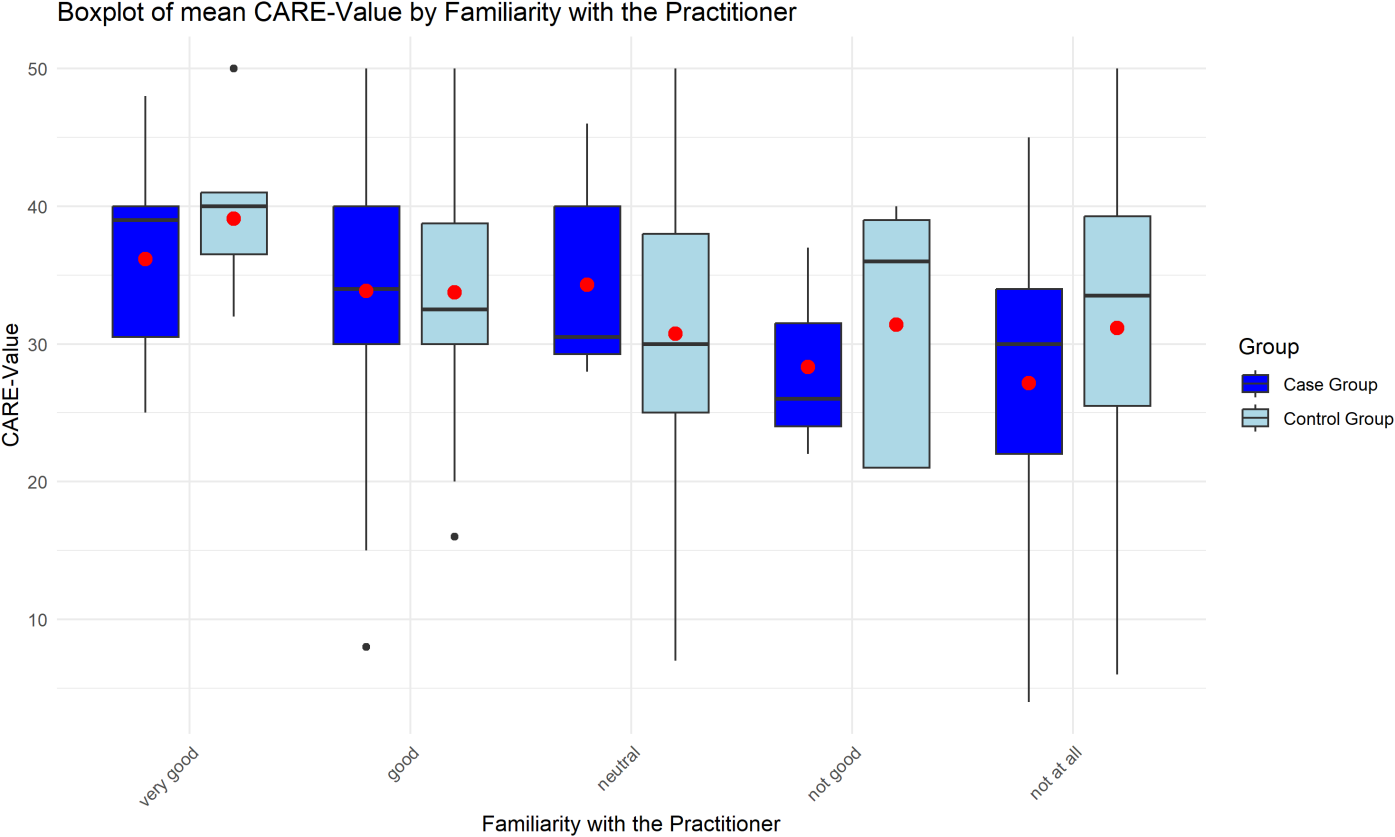
The graph shows a significant correlation between prior familiarity with the clinician and higher CARE scores (p=0.009), indicating improved patient-perceived empathy when the clinician was well-known.

Familiarity with the clinician from previous consultations was significantly associated with higher CARE scores (p = 0.009). Both the case and control groups exhibited higher CARE values when patients reported knowing their clinician “very well.” This relationship is illustrated in **Figure 2**. A Kruskal-Wallis test was performed to analyze CARE scores across different levels of familiarity and group assignments, revealing significant differences (p = 0.0433). *Post-hoc* analysis using a Dunn test showed a significant difference between patients in the case group who were “not at all” familiar with their clinician and those in the control group who were “very well” familiar (p = 0.0147), with an effect size (r = -0.283).

CARE scores also demonstrated a potential relationship with patients’ self-assessed health status. Patients who rated their health as “very good” reported the highest CARE values; however, this observation did not reach statistical significance (p = 0.10). Due to the small number of patients rating their health as “very poor” (n = 3), these responses were combined with “poor” for the analysis.

The results of the multiple linear regression analysis are summarized in **Table 4**. Among the variables analyzed—health status, tumor stage, recurrence, familiarity with the clinician, clinician gender, consultation duration, and mask usage—only “familiarity with the clinician” was found to be statistically significant (β = 1.73, p = 0.003). None of the other variables had a significant effect, as indicated by p-values exceeding 0.05.

**Table 4:** Multiple linear regression analysis.

Predictors n=161	Regression Coefficient	Confidence Interval	p-value
Health Status in the Last Two Weeks	0.45	-1.20 – 2.09	0,592
Tumor Stage according to UICC	0.09	-3.13 – 3.32	0,954
Recurrence	1.24	-3.28 – 5.77	0,588
Familiarity with the clinician	1.73	0.61 – 2.84	0,003
Gender of the clinician	2.52	-0.99 – 6.03	0,158
Duration of Consultation	1.88	-0.43 – 4.19	0,111
Face Mask	-0.65	3.65 – 2.34	0,667

The regression model (F[7,151] = 2.305, p = 0.029) accounted for 9.65% of the variance in CARE scores, with an adjusted R² of 0.055, indicating a modest explanatory capacity. “Familiarity with the clinician” emerged as the most influential predictor, while the remaining variables, including face mask usage, did not significantly impact CARE scores.

## 4 Discussion

This study investigated the impact of face mask use on the quality of communication and the perceived empathy of clinicians, as assessed from the perspective of patients with oral and maxillofacial tumors. The findings revealed that wearing a face mask during consultations did not significantly influence CARE scores, suggesting that mask use does not impair patients’ perception of clinician empathy. Notably, familiarity with the clinician emerged as the only significant factor positively affecting CARE scores, while tumor stage, recurrence, consultation duration, and clinician gender showed no significant effects. These results indicate that mask-wearing by clinicians does not interfere with perceived communication or empathy and that patients generally accept the use of face masks during consultations. The patient cohort comprised a total of 68 women and 93 men. Similar studies have reported a gender distribution favoring women (39, 40), with a lower mean age compared to our cohort. Additionally, the mean age of the patients was found to be lower in these studies. While Wong et al. (2013) reported the largest patient distribution in the 45-64 age group, Pandya et al. (2022) reported the largest patient distribution in the 50-60 age group (39, 40). The mean age of the patients in the present study was 69 years, with an age range of 34 to 95 years. This demographic profile is consistent with that of other head and neck-cancer patient collectives described in the literature, thereby ensuring the representativeness of the sample (41).

The recurrence rate of oral squamous cell carcinoma in our study was 12%, which is lower than the rates reported in other publications. These other publications reported recurrence rates of 20% (42) or 16% (43). As our study was the inaugural investigation into the correlation between a recurrence situation or high tumor stages of the patients in relation to the mean CARE values, it is not feasible to cite existing literature. The relationship between tumor recurrence, advanced tumor stages, and the use of face masks during consultations remains underexplored. Our findings suggest that patients prioritize effective communication and trust over the visibility of facial expressions, even in advanced disease stages. This indicates that empathy perception is less influenced by face masks and more by the clinician’s verbal communication and demeanor.

While existing literature extensively explores the CARE measure and the effects of face masks independently, few studies combine these topics as our study does. In this context, the aforementioned studies by Wong et al. (2013) and Pandya et al. (2022) stand out due to their methodology (39, 40).

While Wong et al. (2013) identified a significant negative influence of the face mask on the mean CARE value, Pandya et al. (2022) were unable to confirm this significance, a finding that is consistent with our study. The application of a face mask had no discernible impact on the mean CARE scores. It is important to note that Wong et al. (2013) had access to a significantly larger patient population than our study, and that their data set included general medical consultations, which differs from our specialist patient population. Furthermore, Wong et al. (2013) were able to distribute the number of consultations with and without a face mask in a more balanced manner. Pandya et al. (2022) conducted their study in an orthopedic department, which is a more specialized field. The size of the patient population was like that of our study, but the distribution of cases and controls was significantly more heterogeneous. In contrast to our study, patients were only allocated to the case or control group based on their stated preference for or rejection of face masks, rather than based on a consultation with or without face mask. The differences in findings between studies may be attributed to contextual factors, such as the timing of data collection and societal attitudes towards face masks. During the COVID-19 pandemic, mask-wearing became a widely accepted practice associated with infection prevention and safety. This shift in perception may have mitigated potential negative effects of masks on communication and empathy, which were more pronounced in studies conducted prior to the pandemic, when mask-wearing was less common.

While other studies (4, 6, 44) have tested the effects of face masks on emotional and empathic perception with static and decontextualized stimuli, Scheibe et al. (2023) employed a similar methodological approach to that used in our own study (45). The researchers employed dynamic stimuli to examine the correlation between perceived empathy and the use of a face mask. Similarly, this study found that wearing a face mask had no negative impact on perceived empathy. Scheibe et al. (2023) demonstrated that individuals can compensate for missing mouth cues by relying on alternative information channels, such as voice pitch, dynamic eye movement, and verbal content, when provided with dynamic, context-rich information. Therefore, individuals are sufficiently motivated to identify and empathize with their interaction partner if they are provided with sufficient information. We were unable to confirm the previously reported negative impact of wearing a face mask on patients’ empathy towards their doctor, as described in the study by Grundmann et al., 2021 (6). It remains unclear whether this discrepancy is due to differences in study design or the use of the CARE questionnaire as a measurement instrument.

As a potential influencing factor on the mean CARE value, we examined the health status of the patients over the previous two weeks. While Wong et al. (2013) reported a linear correlation between poor health and lower CARE scores, our findings only partially confirmed this correlation (39). Although the highest CARE scores were awarded to patients with a ‘very good’ state of health, this was not a statistically significant finding (p=0.10). Similarly, the study by Mercer et al. (2011) found no significant influence of patient health status on mean CARE score (33).

The factor ‘familiarity with the clinician’ was found to exert a significant influence on the mean CARE value during our study. In both the case and control groups, higher CARE values were assigned if patients indicated that they had a high level of familiarity with their clinician, based on previous consultations. The results suggest that in the control group (no mask), greater familiarity with the clinician improved empathy perception, while in the case group (mask), limited familiarity corresponded to lower CARE scores. These findings align with those of previous studies in the field. In their study, Pandya et al. (2022) report that the factor ‘ clinician familiarity’ was significant and exerted a greater influence on the mean CARE score than wearing a face mask (40). Wong et al. (2013) conducted a more detailed investigation into the interactive effect of wearing a face mask and the factor ‘familiarity with the clinicians’ (39). They also found that patients who already knew their clinician will give better CARE scores overall. However, the wearing of a face mask by the clinician had a greater influence on this group of patients, resulting in a significant reduction in the positive effect of ‘familiarity with the clinician’ and a significant increase in CARE scores.

This, however, gives rise to the question of how patients define their knowledge of a clinician. As Freeman et al. (2002) demonstrate in their study, this topic is inherently complex and does not necessarily correlate with the frequency of previous consultations with the same clinician (46). It is assumed that a good physician-patient relationship is more indicative of a patient’s familiarity with their clinician. To further promote this, the long-term care of patients by a clinician was identified as an essential process (47). Additionally, the factor ‘familiarity with the clinicians’ was found to have a positive effect on patient empowerment, specifically in relation to how patients dealt with their own illness (46, 48).

In contrast to the studies by Fung et al. (2009), Mercer et al. (2011) and Wong et al. (2013), our findings revealed no statistically significant correlation between the duration of the consultation and the mean CARE value (33, 39, 49). Similarly, Pandya et al. (2022) reached the same conclusion (40). Although a longer consultation duration in the case group resulted in higher CARE values, as observed in the study by Wong et al. (2013), this outcome was not replicated in the control group (39). Wong et al. (2013) also observed an increase in the mean CARE value per minute, with a value of 0.32. In other studies longer consultation duration was also found to have no impact on general patient satisfaction (50, 51). In this context, the authors emphasize that the quality of the medical consultation, rather than the duration of the consultation, is the determining factor in patient satisfaction.

Our study found no statistically significant differences in mean CARE scores based on clinician gender, contrasting with other studies reporting higher empathy scores for female clinicians and medical students (52–55). Furthermore, Hojat et al. (2002) demonstrated that surgical disciplines, including oral and maxillofacial surgery, exhibited lower empathy scores compared to other specialties (56). However, it is important to note that the gender distribution of clinicians in our study was significantly skewed towards male clinicians, and that the authors conducted their studies with different empathy scales and without the confounding factor of wearing a face mask.

In the present study, the methodology was consistent with that of previous studies in the comparative literature, which also focused on adult participants (6, 39, 40, 44, 45). Considering the above, it is pertinent to inquire as to the extent to which wearing a face mask affects children’s perception and the factors that may be involved in this process.

Despite the absence of any negative impact on patients’ perceived empathy, our study revealed that only a modest proportion of patients (11%) would prefer their clinician to wear a face mask throughout the entirety of the consultation. Moreover, less than half of the patients considered a mask to be relevant in preventing respiratory infections. In this context, it is necessary to identify the relevant factors influencing the acceptance of a face mask in society and to understand how this acceptance can be increased for possible future pandemics. Various approaches to this topic can be found in the scientific literature.

Although negative attitudes toward face masks are prevalent in the general population, often linked to discomfort and impaired communication (57, 58), our findings reveal that patients who experienced consultations with mask-wearing clinicians did not report such negative effects on perceived empathy or communication. This discrepancy suggests that in the clinical setting, the structured and professional nature of healthcare environments may mitigate concerns commonly associated with mask use, highlighting their protective role without compromising interpersonal interactions. Furthermore it has been demonstrated that individuals are more inclined to utilize a face mask if they are convinced of its efficacy (59–61). It is therefore imperative to enhance public awareness of the efficacy of face masks in preventing infection. It can be posited that a favorable disposition towards face masks and their efficacy is more likely to encourage individuals to utilize them in their everyday lives (60).

In their respective studies, Lau et al. (2004) and Sim et al. (2014) highlight the pivotal role of individual risk perception in the decision to wear a mask (59, 62). Individuals who perceive themselves to be highly susceptible to infectious diseases are more likely to wear them. The lack of knowledge about a disease can result in some segments of the population failing to recognize the necessity for protective measures (63).

Ensuring equal allocation of clinicians to the case and control groups posed a challenge due to varying duty rosters, rotation schedules, and clinical emergencies. In our clinic, patients are assigned to clinicians based primarily on availability and operational needs. Although efforts were made to minimize familiarity bias, patient preferences may have influenced assignments, and this potential limitation should be considered when interpreting the findings. It should be noted, however, that this study was not artificially constructed; rather, it reflected the actual day-to-day work in the Department of Oral and Maxillofacial Surgery at the University Medical Centre Goettingen.

A further limitation was the established recall system of the outpatient clinic. In the initial two-year period, patients are to be seen at eight-week intervals. In the subsequent three-to-four-month period of the third year, and in the six-month period of the fourth and fifth years, patients are to be seen at these intervals. Furthermore, patients may request to be seen at individually customized, longer intervals after the fifth year. While a considerable number of patients could be interviewed daily at the outset of the data collection period, the number of interviews conducted subsequently declined. This discrepancy arose because patients with shorter recall intervals were interviewed only once, while patients with longer recall intervals were excluded entirely due to the limited study timeframe, which spanned from November 2023 to April 2024.

The cancellation of appointments and the death of patients resulted in a further reduction in the patient population. A total of 161 patients were interviewed. To ensure the reliability of the results, further research should be conducted at other clinics with a larger case and control group in the future.

In contrast to the findings of other studies (39, 40), this study made a distinction between consultations conducted by female and male clinicians. Furthermore, achieving an even gender distribution of consultations presented a challenge in this context. Most consultations were conducted by male clinicians. However, this reflects the current gender distribution among the Department of Oral and Maxillofacial Surgery at the University Medical Center Goettingen. Furthermore, the exclusion criteria and the collective were reduced by the presence of language barriers or physical impairments of the patients (e.g. vision loss, hearing loss, writing difficulties).

## 5 Conclusion

The wearing of a face mask had no significant impact on physician-patient communication or the perceived empathy of the clinician. Neither tumor recurrence nor advanced tumor stages significantly influenced patients’ empathy ratings, likely because verbal communication and trust-building play a greater role than non-verbal cues like facial expressions. The only significant factor influencing mean CARE scores was patients’ familiarity with their clinician.

It is of the utmost importance to learn from past experiences to enhance preparedness for future pandemics.

Our findings indicate that face masks worn by clinicians do not negatively affect empathy as perceived by patients. However, only a limited proportion of patients expressed a preference for clinicians to wear a face mask throughout consultations. This may reflect factors such as comfort, communication preferences, or a general reluctance toward mask use. While we did not specifically assess patients’ knowledge of mask efficacy, these results emphasize the need for clear communication regarding the protective role of masks in preventing infections. To promote mask acceptance in clinical practice, strategies should focus on building trust within the clinician-patient relationship and ensuring safety in the clinical setting. Increased societal support for mask use in healthcare settings, particularly during future public health crises, may further enhance their acceptance and effectiveness.

## Data Availability

The raw data supporting the conclusions of this article will be made available by the authors, without undue reservation.
